# The Association of Aberrant Expression of FGF1 and mTOR-S6K1 in Colorectal Cancer

**DOI:** 10.3389/fonc.2021.706838

**Published:** 2021-09-06

**Authors:** Tinghui Duan, Diyuan Zhou, Yizhou Yao, Xinyu Shao

**Affiliations:** ^1^ Department of Medical Imaging, The Affiliated Guangji Hospital of Soochow University, Suzhou, China; ^2^ Department of General Surgery, The First Affiliated Hospital of Soochow University, Suzhou, China; ^3^ Department of Gastroenterology, The Affiliated Suzhou Hospital of Nanjing Medical University, Suzhou Municipal Hospital, Gusu School, Nanjing Medical University, Suzhou, China

**Keywords:** colorectal cancer, FGF1, mTOR-S6K1 pathway, prognosis, survival

## Abstract

Colorectal cancer (CRC) is one of the most frequent malignant neoplasms worldwide, and the effect of treatments is limited. Fibroblast growth factor 1 (FGF1) has been involved in a wide variety of several malignant diseases and takes part in the tumorigenesis of CRC. However, the function and mechanism of FGF1 in CRC remains elusive. In this study, the results indicated that FGF1 is elevated in CRC tissues and linked with poor prognosis (*P* < 0.001). In subgroup analysis of FGF1 in CRC, regardless of any clinic-factors except gender, high level FGF1 expression was associated with markedly shorter survival (*P* < 0.05). In addition, the expression of p-S6K1 and FGF1 was not associated in normal tissue (*P* = 0.781), but their expression was closely related in tumor tissue (*P* = 0.010). The oncogenic role of FGF1 was determined using *in vitro* and *in vivo* functional assays. FGF1 depletion inhibited the proliferation and migration of CRC cells *in vitro* and *vivo*. FGF1 was also significantly correlated with mTOR-S6K1 pathway on the gene and protein levels (*P* < 0.05). In conclusion, FGF1 acts as a tumor activator in CRC, and against FGF1 may provide a new visual field on treating CRC, especially for mTORC1-targeted resistant patients.

## Introduction

Colorectal cancer (CRC) is the second cause of cancer death in western countries. Nearly half of the CRC patients will die from the disease which is due to distant metastasis of the primary CRC (1, 2). CRC is also one of the most prevalent malignant tumors in China, with high incidence rate and mortality. Early screening has been shown to be effective in reducing the incidence of, and mortality from CRC. A curative therapy to control the huge threat should include surgical and nonsurgical treatment. Thus, it is extremely urgent to identify effective biomarker for diagnosis and against target for treatment.

Surrounding non-neoplastic stroma acts an important role in the metastasis and invasion of tumor. Stromal cells, at the front of malignant tumor invasion, have a complicated interaction with tumor cells. Fibroblast growth factors (FGFs), called a family of heparin-binding growth factors, interact with several kinds of endothelial receptors which leads to the angiogenesis (3–5). It is recognized that the invasion and metastasis of tumor have something to do with angiogenesis, also numerous studies show the progress of inducing angiogenesis.

FGFs are closely related to FGF receptors (FGFRs) on the target cells’ surface when their biological activities are exerted. Numerous vitro studies pay their attention on the FGF1 and FGFR1 (4, 6). FGFR1, which helps FGF-1 to exert its biological activity, is expressed by endothelial cells. Various studies measure the expression level of the FGF1 and FGFR1 in different cancers, including breast carcinoma, hepatocellular carcinoma and esophagus cancer. The expression level of FGF1 and FGFR1 in these cancers indicated that FGF1 induce the invasion and metastasis of tumor cells. In cancer cells, amplified FGF1 expression promotes the proliferation and migration ability of tumor cells (7–10). However, its expression level in CRC tissues and its expression correlated with clinical indicators and survival, have not been fully elucidated (11).

In this study, we aim to investigate the expression level of FGF1 in CRC, and detect the relationship between FGF1 expression level and diagnosis and prognosis in CRC patients in subgroup analysis. Meanwhile, we hypothesized that FGF1 regulates CRC development *via* mTOR-S6K1 dependent pathway, and our findings demonstrated a novel role of FGF1 in CRC and identified its potential diagnostic and therapeutic relevance.

## Methods

### Human Tissue Specimens

Pairs of CRC and surrounding normal tissues were collected from 2010 to 2013. None of the patients had received radiotherapy or chemotherapy before radical surgery. Our study has been approved by the Independent Ethics Committee of the Affiliated Suzhou Hospital of Nanjing Medical University and the First Affiliated Hospital of Soochow University (IRB approval number, 2020076), and all patients wrote informed consent.

### Tissue *Specimens* and Immunohistochemistry

Tissues were fixed with 10% formalin, embedded in paraffin, and cut into 5μm-thick sections. Then, they were detected *via* IHC according to the protocol as our previous study (12, 13). a) The sections were dewaxed in xylene twice and rehydrated using ethanol (95%, 90%, 75%) and distilled water. b) Submerged the sections in sodium citrate antigen-repair buffer and oven heating the buffer to 92-98°C for 15min, and then cool down to room temperature for 2-3 times. Wash with PBS for 2-3 times. c) Block the endogenous peroxidase with 3% hydrogen peroxide for 10-15min, and then washed with PBS for 2-3 times. d) The sections were incubated with 5% goat serum for 30 min, then the goat serum was removed and antibodies were added overnight at 4°C. e) The slides were washed with PBS for 3 times/10 min and incubated with secondary antibodies for 30 min, and then washed with PBS for 3 times/10 min. f) Finally, the sections were colored with diaminobenzidine (DAB) and stained nuclei with hematoxylin. g) Seal the sections with neutral resins.

A tissue staining kit (Zhongshan Biotechnology, China) was used and tissue sections were incubated overnight with 1:200 diluted polyclonal anti-human FGF1 (BOSTER, China) or anti-human p-S6K1 (Cell Signaling Technology, USA) at 4°C. The percentage of positively stained cells was scored as follows: score 0 represents 0-5%; score 1 represents 6-25%; score 2 represents 26-50%; score 3 represents 51-75%; score 4 represents >75%. The staining intensity was scored as 0 (negative), 1 (weak), 2 (moderate) and 3 (strong). The percentage score was multiplied by intensity score to show the selected region. The final score was the average of five random selected regions. It graded as follows: – (0), + (1-4), ++ (5-8) and +++ (9-12). Samples with final scores ++ or +++ were graded positive, and - or + as negative. The tumor budding was quantified according the criteria of International Tumor Budding Consensus Conference (ITBCC) (14). The invasion front and hot-spot areas were identified in low-power view, then single cell and clusters of up to 4 cells at the invasive margin of CRC were counted with 20 x objective lens (15, 16).

### Bioinformatics Analysis

Relevant gene expression datasets were analyzed *via* the Oncomine (https://www.oncomine.org) and GEPIA (Gene Expression Profiling Interactive Analysis, http://gepia.cancer-pku.cn) platform.

### Cell Culture and Transfection

CRC cell lines were purchased from the Cell Bank of Chinese Academy of Sciences (Shanghai, China), and were cultured in RPMI 1640 medium (Hyclone, USA) supplemented with 10% fetal bovine serum (Gibco, USA), penicillin G sodium (100U/ml) and streptomycin (100μg/ml) at 37°C under 5% CO_2_. The cells were grown till 70% confluency, and transfected with human FGF1 shRNA (5’-CCGGGCCCTGACCGAGAAGTTTAATTTCAAGAGAATTAAACTTCTCGGTCAGGGCTTTTTT-3’) according to the manufacturer’s instructions. The transfected cells were selected using 500μg/ml G418 (Roche, Switzerland) for 3-4 weeks, and clones with a stable knockdown of FGF1 were selected for further experiments.

### RNA Isolation and Quantitative Real-Time PCR

Total RNA was extracted from the tissues or cells using TRIzol reagent (Invitrogen, Life Technologies, USA) according to the manufacturer’s protocol. Following DNAse I (Thermo Fisher Scientific, USA) treatment to remove genomic DNA, 1μg RNA was reverse transcribed using a RevertAid First Strand cDNA Synthesis Kit (Thermo Fisher Scientific, USA). The qRT-PCR was performed using Power SYBR^®^ Green PCR Master Mix (ABI, USA) on the 7500 real time PCR system (ABI, USA) according to the manufacturer’s instructions. The following primers were used: FGF1 forward (5’-GTGGATGGGACAAGGGACAG-3’) and reverse (5’-GGCAGGGGGAGAAACAAGAT-3’); β-actin forward (5’- CCACACTGTGCCCATCTACG-3’) and reverse (5’-AGGATCTTCATGAGGTAGTCAGTCAG-3’). Fold changes were calculated relative to β-actin (internal control) using the 2^-ΔΔC^T method.

### Western Blotting

The extracted proteins were separated by SDS-PAGE and transferred onto PVDF membranes (Millipore, USA). After blocking with 5% non-fat milk for 1h, the membranes were probed overnight with FGF1 (1:1000, BOSTER, China), p-mTOR (1:1000, Cell Signaling Technology, USA), mTOR (1:1000, Bioss, China), p-S6K1 (1:1000, Cell Signaling Technology, USA), and S6K1 (1:1000, Bioss, China) and β-Actin (1:5000, BOSTER, China) antibodies at 4°C with gentle shaking, followed by horseradish peroxidase-conjugated secondary antibodies. The protein bands were visualized by chemiluminescence and quantified by ImageJ (NIH, USA).

### Colony Formation Assay

The suitably transfected cells were seeded in 6-well plates at the density of 1000 cells/well, and cultured for 10 days before being fixed and stained with 0.1% crystal violet. The colonies with more than 100 cells were counted at 40x magnification under an optical microscope (Nikon, Japan) fitted with a digital camera (Nikon, Japan).

### Transwell *Assay*

Cell migration ability was assessed using Transwell inserts (pore size 8μm; Corning, USA). The cells were seeded into the upper chambers of the inserts at the density of 10,000 cells/200µl in serum-free RPMI 1640 medium, and the lower chambers were filled with 800μl complete medium per well. After incubating for 12h at 37°C, the cells remaining on the upper surface of the membrane were removed using a cotton swab. The filters were then fixed with 4% paraformaldehyde, and the cells on the lower surface were stained with 0.1% crystal violet and counted in 5 random fields per sample.

### Wound Healing Assay

Wound healing assay was also adopted to test the migration ability of colon cancer cells. Cells were plated in 6-well plates, when cell confluence reached approximately 100%, the old medium was removed and the monolayer was wounded by scratching with a 100μl sterile pipette tip lengthwise along the chamber, then cells were washed three times with PBS and cultured with serum-free medium at 37°C. Images of cells migrating into the wound were recorded at 0h and 24h using an inverted microscope. Wound width was measured using OpenLab (Agilent, USA). The experiments were repeated three times.

### Subcutaneous Xenograft Establishment

SPF male BALB/c nude mice (4weeks old and weighing 16-18g) were purchased from Shanghai SLRC laboratory Animal Co. Ltd. (Shanghai, China). The mice were randomly divided into the FGF1 knock down (KD) and negative control (NC) groups (n = 5 per group), and accordingly injected subcutaneously with 5×10^6^ FGF1-KD or NC-shRNA HCT116 into the right dorsal flank on day 0. All animal experiments were approved by the Animal Ethics Committee of the Affiliated Suzhou Hospital of Nanjing Medical University (Suzhou, China).

### Statistical *Analysis*

All methods were carried out in accordance with relevant guidelines and regulations. All patients were followed up by personal or telephonic interviews for 60 months, and the time point was set as the date of CRC-related death or 60 months after surgery. Self-developed R program (version 3.6.1 for Windows, http://cran.r-project.org/) was used for Cluster analysis and Nomogram analysis. SPSS 22.0 software (SPSS Inc., Chicago, IL, USA) and GraphPad Prism 8 (San Diego, CA) were also used to perform statistical analysis. All data were presented as mean ± SD of three independent experiments. The Student’s t-test (unpaired, two-tailed) or one-way ANOVA were used to compare means between two groups. IHC results were analyzed by Chi-squared or Fisher’s exact tests. *P* < 0.05 was considered statistically significant.

## Results

### FGF1 Is Elevated in CRC Tissues and Linked With Poor Prognosis

**Figure 1:**
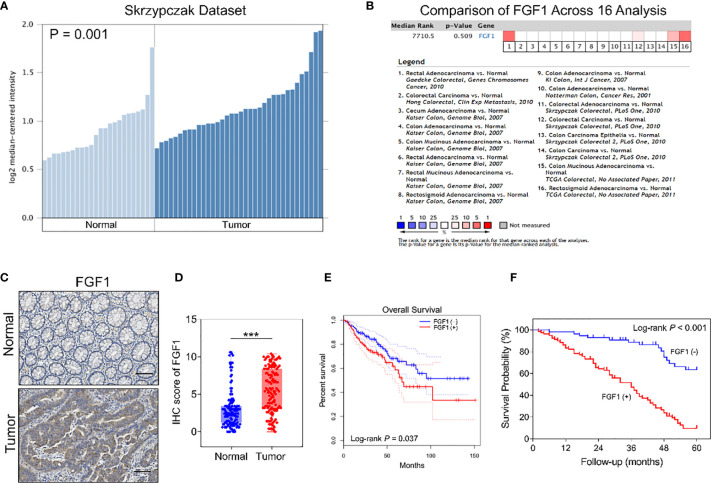
Expression of FGF1 in CRC and paired normal tissues. **(A)** FGF1 mRNA levels in CRC and normal tissues in Skrzypczak’s datasets. **(B)** Comparison of FGF1 mRNA expression in CRC and normal tissues across 16 Oncomine datasets. **(C)** Representative IHC images showing *in situ* FGF1 expression in CRC and normal tissues (scale bar = 100μm). **(D)** IHC scores of FGF1 in CRC vs normal tissues. **(E)** OS of FGF1(+) and FGF1(-) in CRC patients in TCGA dataset searched *via* GEPIA platform. **(F)** OS of FGF1(+) and FGF1(-) in CRC patients. ***P < 0.001.

To assess the FGF1 expression level in colorectal normal tissue and paired CRC tissue, we searched the findings of Skrzypczak et al, which revealed FGF1 was aberrant active in CRC tissue (**Figure 1A**). However, the outcome was not consistent with the findings of pooled analysis of FGF1 expression of CRC and normal tissues across 16 datasets searched *via* Oncomine platform (**Figure 1B**). To confirm the inconsistent result, we detected the FGF1 expression in 135 CRC and paired normal colorectal tissues *via* IHC. The FGF1 protein was reduced in normal colon tissues, and significantly higher in the CRC tissues (*P* < 0.001, **Figures 1C, D** and **Table 1**).

**Table 1:** Statistics of FGF1 expression in 135 CRC tissues and adjacent normal tissue.

	FGF1	
	Positive	Negative
Tumor tissue	79	56
Normal tissue	21	114
c^2^	53.428	
*P* value	<0.001	

Moreover, FGF1 expression was significantly associated with lymph node metastasis (*P* = 0.003), tumor budding degree (P = 0.002) and TNM stage (P = 0.001, **Table 2**), while no correlation was observed with other clinicopathological variables such as age, gender, tumor size, the depth of invasion, degree of differentiation, venous invasion and neural invasion (*P* > 0.05, **Table 2**). In further univariate analysis, it revealed that depth of invasion, degree of differentiation, lymph node metastasis, venous invasion, neural invasion, TNM stage and FGF1 expression (*P* < 0.001, **Table 3**) acted as an independent prognostic factor for the survival of CRC patients. Meanwhile, FGF1 expression level also played a significant role in multivariate analysis (*P* < 0.001, **Table 3**). To demarcated the patients according to FGF1 expression levels, we found the reduced FGF1 expression promoted prognosis in TCGA dataset searched by GEPIA platform (**Figure 1E**) and checked in 135 CRC tissues (**Figure 1F**).

**Table 2:** Relationship between FGF1 and clinic-pathological factors in CRC patients.

Variables	FGF1		
	Negative	Positive	*P* value
Age (years)			
≤60	19	38	0.100
>60	37	41	
Gender			
Male	32	45	0.983
Female	24	34	
Tumor size (cm)			
<5	27	32	0.374
≥5	29	47	
Depth of tumor invasion			
T1-2	14	11	0.103
T3-4	42	68	
Lymph node metastasis			
No	36	30	0.003*
Yes	20	49	
Degree of differentiation			
Well	48	63	0.372
Poor	8	16	
Venous invasion			
Negative	44	52	0.107
Positive	12	27	
Neural invasion			
Negative	42	49	0.113
Positive	14	30	
Tumor budding degree			
Low	36	29	0.002*
High	20	50	
TNM staging			
I-II	36	28	0.001*
III-IV	20	51	

*P < 0.05.

**Table 3:** Results of univariate and multivariate analyses of underwent gastrectomy patients’ survival by Cox’s proportional hazard model.

Varieties	n	Univariate analysis			Multivariate analysis		
		HR	95% CI	*P*	HR	95% CI	*P*
Age (≤60 or >60 years)	57/78	1.084	0.696-1.687	0.722			
Gender (Male/Female)	77/58	0.876	0.561-1.366	0.559			
Tumor size (≤5 or >5 cm)	59/76	0.654	0.418-1.023	0.063			
Depth of tumor invasion (T1-2/T3-4)	25/110	0.223	0.102-0.487	<0.001*	0.350	0.154-0.794	0.012*
Lymph node metastasis (negative/positive)	66/69	0.179	0.108-0.298	<0.001*	9.250	1.892->9.000	0.006*
Degree of differentiation (well or poor)	111/24	0.461	0.270-0.787	0.005*	0.639	0.365-1.118	0.117
Venous invasion (negative/positive)	96/39	0.374	0.236-0.595	<0.001*	0.618	0.374-1.022	0.061
Neural invasion (negative/positive)	91/44	0.544	0.346-0.856	0.008*	0.880	0.541-1.431	0.607
TNM staging (I-II/III-IV)	64/71	0.157	0.093-0.264	<0.001*	0.029	0.006-0.149	<0.001*
FGF1 expression (negative/positive)	56/79	0.188	0.107-0.332	<0.001*	0.204	0.113-0.371	<0.001*

*P < 0.05.

### Subgroup Analysis of FGF1 in CRC

**Figure 2:**
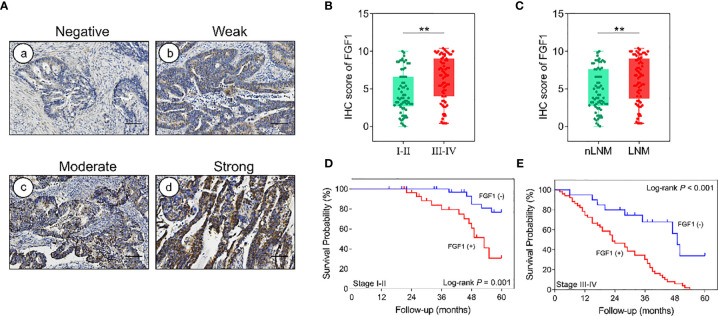
Effect of FGF1 on survival. **(A)** IHC images showing *in situ* FGF1 expression in CRC tissues (scale bar = 100μm). Negative (a), Weak (b), Moderate (c), Strong (d). **(B)** IHC scores of FGF1 in TNM stage I-II vs stage III-IV. **(C)** IHC scores of FGF1 in nLNM vs LNM. **(D, E)** OS of FGF1(+) and FGF1(-) CRC patients with TNM staging **(D)** I-II and **(E)** III-IV. nLNM, no lymph node metastasis; LNM, lymph node metastasis. **P < 0.01.

After the analysis of FGF1 expression in CRC and normal tissues, and expression effect on prognosis, we further investigated the subgroup influence of FGF1 exerts in CRC. 135 CRC tissues were assessed according to the outcome of IHC test (**Figure 2A**). FGF1 levels was significantly increased in patients with tumor−node−metastasis (TNM) stage III-IV compared with I-II (**Figure 2B**). Furthermore, FGF1 expression showed similar elevated trend in CRC tissues with lymph node metastasis compared with those without *in situ* (**Figure 2C**). No matter in TNM stage I-II or III-IV subgroup, amplified FGF1 expression still indicated the poor prognosis (**Figures 2D, E**).

**Figure 3:**
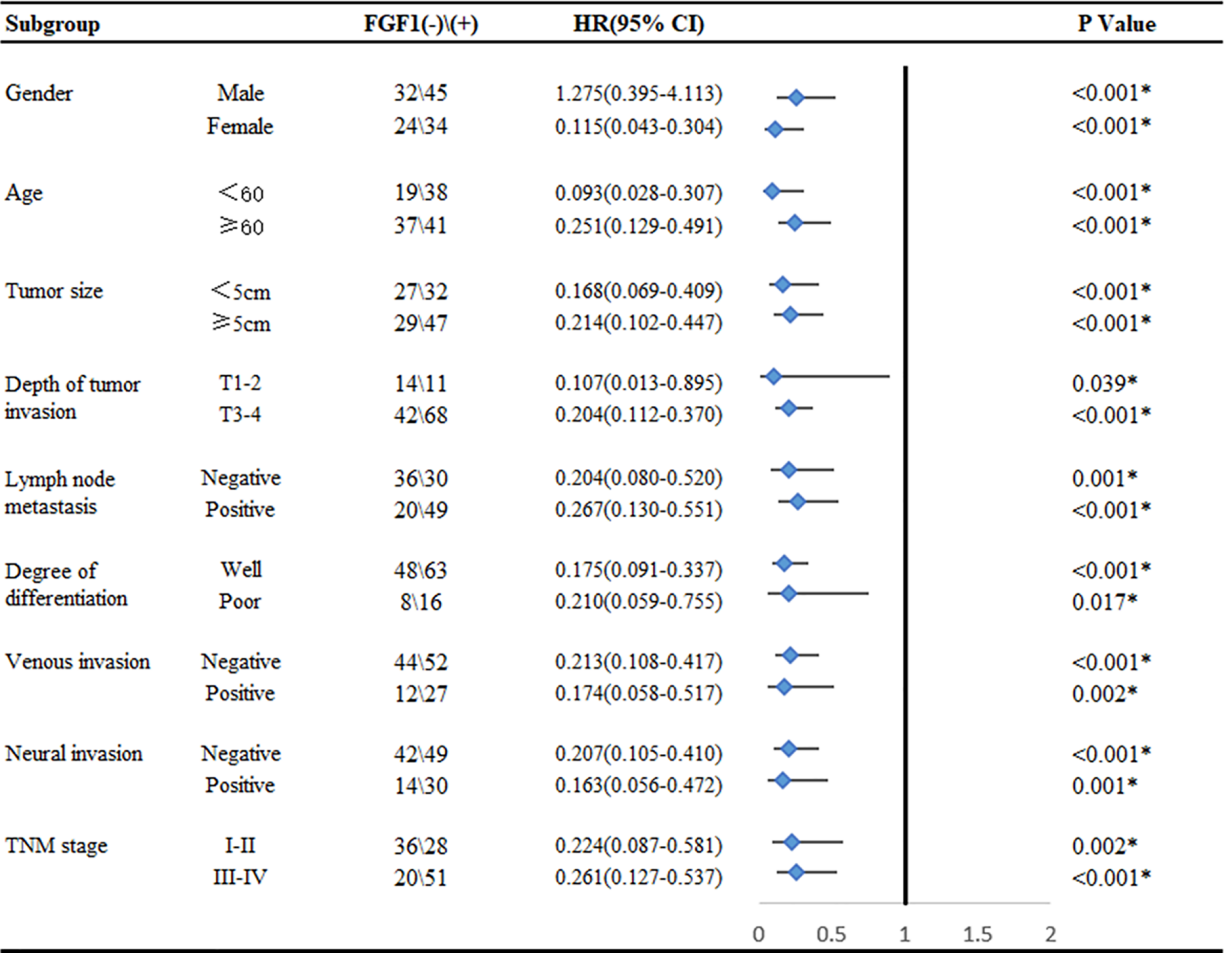
Subgroup analysis for the influence factor of survival duration of CRC patients according to FGF1 expression. *P < 0.05.

In order to better reveal the influence of FGF1 level on the prognosis of CRC patients, we conducted subgroup analysis on prognosis of patients based on various clinicopathological characteristics. The outcome suggested that regardless of age, gender, tumor size, depth of invasion, lymph node metastasis, degree of differentiation, venous invasion, neural invasion and TNM stage, high level FGF1 expression was associated with markedly shorter survival (**Figure 3**).

### The Nomogram Predicts the Prognosis of the CRC Patients

**Figure 4:**
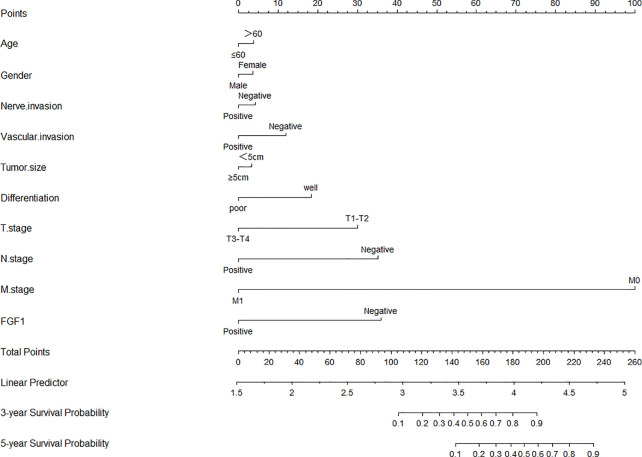
Nomograms to predict survival of CRC patients. Points of each variable were obtained *via* a vertical line between each variable and the point scale. The predicted survival rate was correlated with the total points by drawing a vertical line from the total points scale to the overall survival.

Age, gender, neural invasion, vascular invasion, tumor size, differentiation, T stage, N stage, M stage and FGF1 expression were used to estimate 3- and 5- year OS (**Figure 4**). The nomogram gave every prognostic variable a score on the point scale and we found a score associated with each prognostic factor on the nomogram point scale and calculated the total score (**Figure 4**). And, FGF1 acted as an important role in the prognosis of CRC patients.

### Association Between FGF1 and p-S6K1 Expression

**Figure 5:**
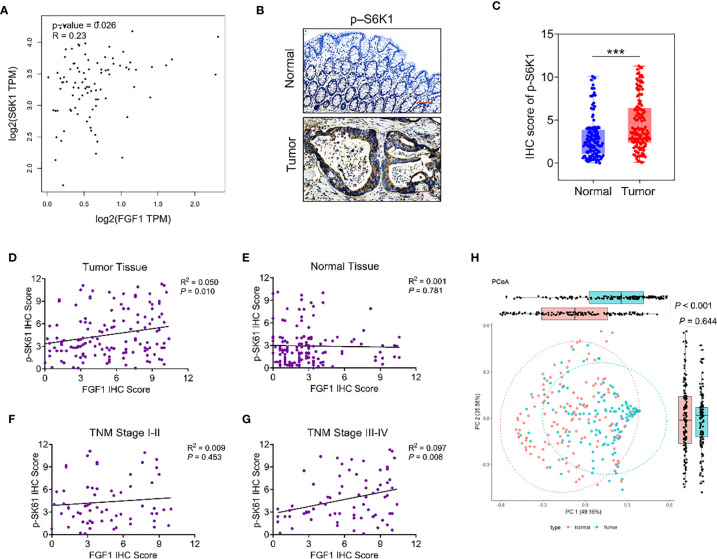
The relationship between FGF1 and p-S6K1.Nomograms to predict survival of CRC patients. **(A)** Correlation analysis of FGF1 and S6K1 at gene level in CRC tissues from TCGA datasets by GEPIA platform. **(B)** Representative IHC images showing *in situ* p-S6K1 expression in CRC and normal tissues (scale bar = 100μm). **(C)** IHC scores of p-S6K1 in CRC vs normal tissues. **(D–G)** Correlation between FGF1 and p-S6K1 at protein level in **(D)** CRC tissue, **(E)** normal tissue, **(F)** TNM stage I-II tissue and **(G)** TNM stage III-IV tissue. **(H)** Stratification of 135 pairs of CRC and normal tissues into cluster 1 and cluster 2 according to FGF1 and p-S6K1 IHC scores. ***P < 0.001.

As reported, studies suggested that FGF1 may promote proliferation and metastasis of tumor cells by regulating the AKT-mTOR-S6K1 signaling pathway in a variety of tumors. TCGA dataset analysis by GEPIA platform showed that the expression of FGF1 in colorectal cancer tissues was positively correlated with S6K1 (**Figure 5A**). To verify the relationship between FGF1 and the AKT-mTOR-S6K1 axis in CRC, firstly, we investigated the expression of p-S6K1 in 135 samples in protein level *via* IHC (**Figure 5B**). Compared to normal tissues, the expression of p-S6K1 in CRC tissue was significantly elevated (**Figure 5C**). Then, we examined the correlation between FGF1 and p-S6K1 expression. The expression of p-S6K1 and FGF1 was not associated in normal tissue, but their expression was closely related in tumor tissue (**Figures 5D, E**).

In subgroup analysis according to the TNM stage, their expression was no obvious association in tissues at TNM stage I-II (**Figure 5F**). Whereas, in the TNM stage III-IV group, p-S6K1 was closely linked to FGF1 (**Figure 5G**). Therefore, the expression of FGF1 and p-S6K1 increased aberrantly in a large proportion of CRC specimens. Meanwhile, p-S6K1 expression was associated with FGF1 expression obviously in advanced CRC. Moreover, PCoA cluster analysis was performed based on the IHC scores of FGF1 and p-sk61. The difference between tumor tissue and normal tissue can be clearly distinguished on the PC1 axis (**Figure 5H**).

**Figure 6:**
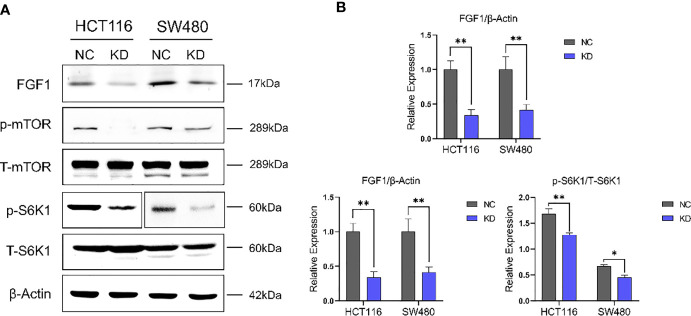
FGF1 regulates CRC cell growth in an mTOR-S6K1 pathway dependent manner. **(A)** Immunoblot showing FGF1, p-mTOR, mTOR, p-S6K1 and S6K1, β-Actin protein levels in CRC cells transfected with FGF1-shRNA. **(B)** Immunoblot result of FGF1/β-Actin, p-mTOR/mTOR and p-S6K1/S6K1 were semi-quantified by ImageJ. Data are presented as mean ± SD. NC, negative control; KD, FGF1-shRNA. *P < 0.05, **P < 0.01.

To gain further mechanistic insights, we examined the knockdown effect of FGF1 in colorectal cancer cells firstly. Then, the p-mTOR and p-S6K1 expression in CRC cells transfected with FGF1-shRNA was analyzed. It indicated that inhibiting FGF1 downregulated mTOR-S6K1 pathway (**Figures 6A, B**).

### Ectopic FGF1 Promotes Proliferation and Migration Ability of CRC Cells

**Figure 7:**
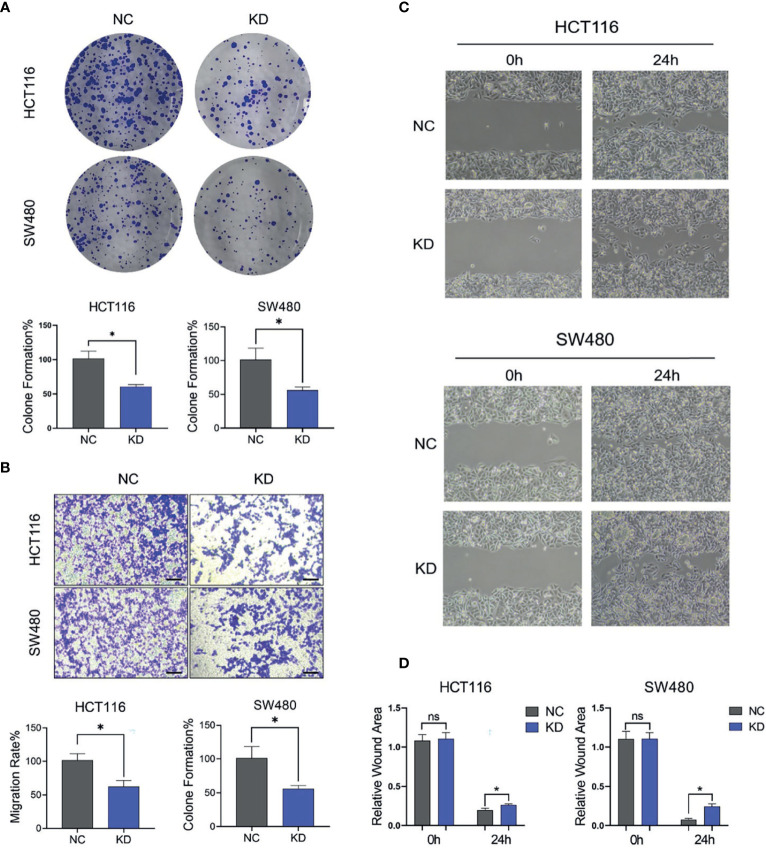
FGF1 promotes proliferation and migration ability of CRC cells. **(A, B)** Colony formation capacity **(A)** and migration rates **(B)** of FGF1-KD CRC cells. CRC, colorectal cancer. **(C)** Wound healing assays were carried out at 24h after transfection in 6-well plates. The gap width was measured using Open Lab software. **(D)** The wound rate was calculated and displayed graphically according to the measured results by Open Lab software. NC, negative control; KD, FGF1-shRNA. Data are presented as mean ± SD (n=3). Ns, no significance, *P < 0.05.

mTOR-S6K1 pathway is closely related to the proliferation and migration of cancer cells. Due to the close regulatory relationship between FGF1 and mTOR-S6K1 pathway, we investigated the biological role of FGF1 on the proliferation and migration ability of CRC cells. Compared to the negative controls, the colony-formation ability of FGF1-KD cells was significantly reduced (**Figure 7A**). Consistent with this, the FGF1-KD cells also showed decreased migration ability in transwell assays (**Figure 7B**), and wound healing assays (**Figures 7C, D**). Taken together, the ectopic expression of FGF1 promotes tumorigenesis of CRC cells *in vitro*.

### FGF1 Enhances Colorectal Tumorigenesis *In Vivo*

**Figure 8:**
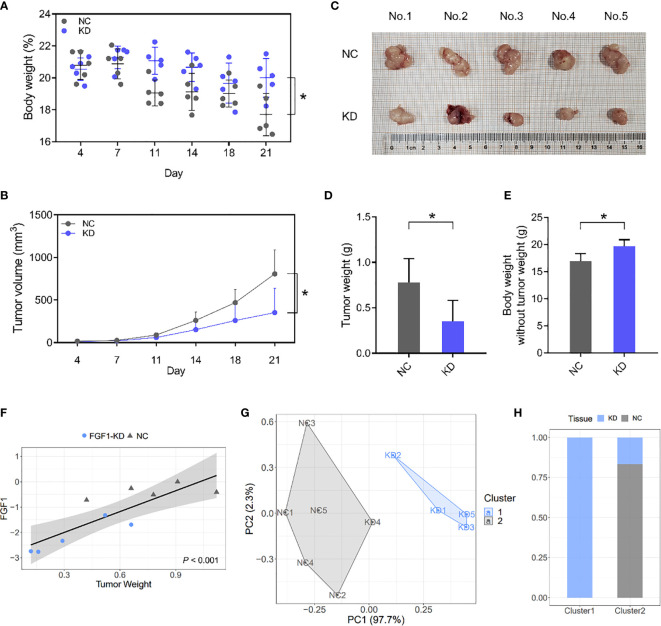
FGF1 induces CRC tumor growth *in vivo*. **(A, B)** Total body weight **(A)** and tumor volume **(B)** of the mice during the experiment. **(C)** Representative pictures of subcutaneous tumors harvested from NC and FGF1-KD group. **(D)** The weights of tumor masses. **(E)** Net body weight after subtracting the respective tumor weights. **(F)** Relative FGF1 mRNA levels in the tumors and the tumor weight correlation. **(G)** Stratification of mice into cluster 1 (blue) and cluster 2 (grey) according to tumor weight and FGF mRNA levels. **(H)** Percentage of NC and FGF1-KD mice in each cluster. Data are presented as mean ± SD (n=5). CRC, colorectal cancer. NC, negative control; KD, FGF1-shRNA. *P < 0.05.

The role of FGF1 in CRC tumor growth was analyzed by establishing an *in vivo* xenograft model using wild-type and FGF1-KD HCT116 cells. Depletion of FGF1 alleviated the nutritional status of mice to a certain extent (**Figure 8A**), and significantly inhibited the proliferative capacity of the CRC cells, which was manifested as reduced tumor size (**Figures 8B, C**) and weight (**Figure 8D**) compared to control group. Then, we evaluated the difference of mice weight after tumor removal, and the weight of mice in FGF1-KD group was still superior to negative controls (**Figure 8E**).

Furthermore, FGF1 mRNA levels *in situ* were markedly affected the weight of Subcutaneous tumor, and showed significant statistical correlation (**Figure 8F**). We next performed a cluster analysis to consider the combined effects of tumor volume and FGF1 expression level (**Figure 8G**). 80% of the FGF1-KD group mice were in Cluster 1, and 100% of the NC and 20% of the FGF1-KD group mice were in Cluster 2 (**Figure 8H**). It suggests that there is a significant difference between FGF1-KD and negative control CRC cells *in vivo.*

## Discussion

In recent years, at least 22 different FGFs have been identified, ranging from nematodes and fruit flies to mice and humans (17, 18). FGF family members possess broad mitogenic and cell survival activities, and are involved in a variety of biological processes, including embryonic development, cell growth, morphogenesis, tissue repair, tumor growth and invasion. As an important member of the FGFs family, FGF1 functions as a modifier of endothelial cell migration and proliferation, as well as an angiogenic factor (5, 7). It acts as a mitogen for a variety of mesoderm- and neuroectoderm-derived cells *in vitro*, thus is thought to be involved in organogenesis.

As reported, FGF2 and FGF20 are the major FGFs involved in embryogenesis and colorectal tissue regeneration. However, the expression level and role of FGF1 in colorectal cancer remain unclear. In this study, FGF1 expression was significantly higher in colorectal cancer than in normal tissues by detecting 135 normal and paired CRC tissues. Regarding the detection of FGF1 expression level, our results were consistent with Skrzypczak’s (19). However, a pooled analysis of 16 studies included by the Oncomine platform do not support the result that expression levels of FGF1 is higher in CRC than in normal tissues (20, 21). Therefore, more studies are needed to clarify and support this conclusion. Meanwhile, elevated FGF1 expression level in tumor tissues was associated with poor prognosis. This result is consistent with TCGA database outcome. Studies in ovarian cancer, breast cancer and lung cancer suggest that FGF1 expression levels in tumor tissues are closely related to prognosis (4, 7–10). However, the prognostic role of FGF1 in CRC patients is still lacking.

In addition to demonstrating that FGF1 predicts a poor prognosis in CRC, we also performed a subgroup analysis to further reveal the association between FGF1 and survival of CRC patients in different subgroups. Interestingly, we found that high expression of FGF1 predicted poor prognosis no matter at different TNM stages, lymph node metastasis, degree of differentiation, and depth of tissue infiltration etc. However, we found different prognostic effects of FGF1 in nongender-specific subgroups. FGF1 was associated with survival in the female subgroup, but not in the male subgroup. It indicates FGF1 may be more effective in predicting survival in female patients. However, this conclusion has its limitation, which may be due to the low number of positive FGF1 detected in CRC tissues in male patients. Therefore, the analysis may not be accurate (22, 23). And, a further increase in male cases is needed to provide reliable analysis.

FGF1 is involved in cell proliferation, survival, migration, invasion, differentiation, and angiogenesis. It is recognized that the invasion and metastasis of tumor have something to do with angiogenesis. As FGF1 was found to be correlated with lymph node metastases. Study suggests that the angiogenesis intensity in CRC is higher in early-stages of the tumoral proliferation. However, it is not an increasing process, having rather an oscillating character (24). High angiogenic activity in early tumor tissues may be an important factor in promoting the gradual increase of FGF1 expression. Thus, it is easily hijacked by cancer cells and shows oncogenic roles in many cancers (17, 18). The phosphorylation of FGFRs by FGFs initiates biological effects through activation of different signaling pathways including the PI3K-AKT-mTOR pathway. However, the association of FGF1 with PI3K-AKT-mTOR pathway in CRC is unclarified (7, 25, 26). TCGA dataset analysis revealed that FGF1 links with SK61, which is the downstream target of mTOR. IHC results showed no correlation between FGF1 and phosphorylated S6K1 in normal colorectal tissues, but a positive correlation in CRC tissues. Furthermore, in a subgroup analysis based on TNM stage, we found no correlation between FGF1 and p-S6K1 in early CRC, while FGF1 was closely associated with p-S6K1 in advanced CRC. This evidence enhanced that FGF1 expression levels and their association with the AKT-mTOR signaling pathway become increasingly tight as tumors develop. And, FGF1 may act as an excellent biomarker to predict the process of tumorigenesis (7).

PI3K-Akt-mTOR is an important metabolic signaling pathway (27, 28), which is aberrant expression in a variety of tumor cells (29–32). Studies have shown that the abnormal activation of mTORC1 complex promotes the proliferation and migration ability of CRC cells (33–35). However, FGF1 is closely related to the activation level of mTORC1, the potential regulatory mechanism between FGF1 and mTORC1 has not been clarified in a variety of digestive tract tumors. To further clarify the regulatory relationship between FGF1 and mTOR related signaling pathways, we knocked down the expression of FGF1, analyzed the expression alteration of mTOR and its downstream targets, and evaluated the proliferation and migration ability of tumor cells *in vitro* and *in vivo*. The results indicated that knockdown of FGF1 negatively regulated the phosphorylation levels of mTOR and S6K1. Meanwhile, down-regulation of FGF1 expression can effectively inhibit the proliferation and migration ability of colorectal cancer cells *in vitro*. *In vivo*, down-regulation of FGF1 expression delayed the occurrence and progression of tumors and effectively improved the nutritional status of tumor-bearing mice.

In sum, there are still few studies on FGF1 in CRC, and its role and underlying mechanism have not been fully elucidated. The comprehensive understanding of FGF1 structure and function, as well as elucidation of the specific FGF1 inhibitor interactions, and combined with further study of the effect of FGF1 in CRC may promote new strategies for the treatment of CRC.

## Data Availability Statement

The original contributions presented in the study are included in the article/supplementary material. Further inquiries can be directed to the corresponding authors.

## Ethics Statement

The studies involving human participants were reviewed and approved by the Ethics Committee of the Affiliated Suzhou Hospital of Nanjing Medical University. The patients/participants provided their written informed consent to participate in this study. The animal study was reviewed and approved by the Animal Ethics Committee of the Affiliated Suzhou Hospital of Nanjing Medical University.

## Author Contributions

TD and XS carried out the literature search, experiments and data acquisition, participated in the study design, and drafted the manuscript. TD, DZ, YY, and XS collected the data, participated in the study design, and helped draft the manuscript. All authors contributed to the article and approved the submitted version.

## Conflict of Interest

The authors declare that the research was conducted in the absence of any commercial or financial relationships that could be construed as a potential conflict of interest.

## Publisher’s Note

All claims expressed in this article are solely those of the authors and do not necessarily represent those of their affiliated organizations, or those of the publisher, the editors and the reviewers. Any product that may be evaluated in this article, or claim that may be made by its manufacturer, is not guaranteed or endorsed by the publisher.
