# Corrigendum: The Association of Aberrant Expression of FGF1 and mTOR-S6K1 in Colorectal Cancer

**DOI:** 10.3389/fonc.2021.792453

**Published:** 2021-10-25

**Authors:** Tinghui Duan, Diyuan Zhou, Yizhou Yao, Xinyu Shao

**Affiliations:** ^1^Department of Medical Imaging, The Affiliated Guangji Hospital of Soochow University, Suzhou, China; ^2^Department of General Surgery, The First Affiliated Hospital of Soochow University, Suzhou, China; ^3^Department of Gastroenterology, The Affiliated Suzhou Hospital of Nanjing Medical University, Suzhou Municipal Hospital, Gusu School, Nanjing Medical University, Suzhou, China

**Keywords:** colorectal cancer, FGF1, mTOR-S6K1 pathway, prognosis, survival

In the original article, there was a mistake in ***Figure 7B*** as published. We identified a minor error in image-misusing was made. The corrected ***Figure 7*** appears below.

**Figure 7:**
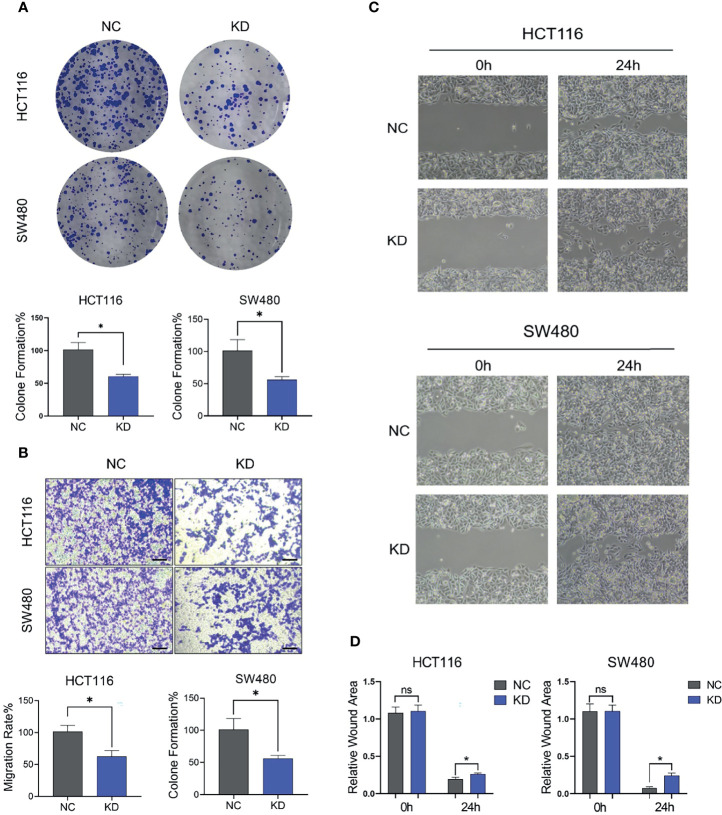
FGF1 promotes proliferation and migration ability of CRC cells. **(A, B)** Colony formation capacity **(A)** and migration rates **(B)** of FGF1-KD CRC cells. CRC, colorectal cancer. **(C)** Wound healing assays were carried out at 24h after transfection in 6-well plates. The gap width was measured using Open Lab software. **(D)** The wound rate was calculated and displayed graphically according to the measured results by Open Lab software. NC, negative control; KD, FGF1-shRNA. Data are presented as mean ± SD (n=3). Ns, no significance, *P < 0.05.

The authors apologize for this error and state that this does not change the scientific conclusions of the article in any way. The original article has been updated.

## Publisher’s Note

All claims expressed in this article are solely those of the authors and do not necessarily represent those of their affiliated organizations, or those of the publisher, the editors and the reviewers. Any product that may be evaluated in this article, or claim that may be made by its manufacturer, is not guaranteed or endorsed by the publisher.

